# Life-Stage and Parasite-Induced Shifts in the Skin Mucus Proteome of Nile tilapia (*Oreochromis niloticus*)

**DOI:** 10.3390/biology15201864

**Published:** 2026-10-16

**Authors:** Ning Yan, Rui Sun, Jingyu Zhuang, Qianyu Lu, Jie Cheng, Caijun Xiong, Jinyun Liu, Xinyue Jiang, Shengfeng Huang, Jinsong Chen

**Affiliations:** State Key Laboratory of Biocontrol, Southern Marine Science and Engineering Guangdong Laboratory (Zhuhai), Guangdong Provincial Key Laboratory for Aquatic Economic Animals, School of Life Sciences, Sun Yat-sen University, Guangzhou 510275, China; yann6@mail2.sysu.edu.cn (N.Y.); sunruiyes@gmail.com (R.S.); 15917360434@163.com (J.Z.); luqy39@mail2.sysu.edu.cn (Q.L.); 13625539443@163.com (J.C.); xcj2721855215@163.com (C.X.); liujy585@mail2.sysu.edu.cn (J.L.); jiangxy263@mail2.sysu.edu.cn (X.J.); hshengf2@mail.sysu.edu.cn (S.H.)

**Keywords:** *Oreochromis niloticus*, parasite, *Cichlidarus cichlidarum*, skin mucus, immunity

## Abstract

Nile tilapia (*Oreochromis niloticus*) skin mucus serves as a critical primary defense against aquatic pathogens, yet its developmental and infection-induced proteomic dynamics remain poorly characterized. This study utilized liquid chromatography–mass spectrometry (LC-MS)-based comparative proteomics to characterize skin mucus profiles across developmental stages and during monogenean *Cichlidarus cichlidarum* infection. The analysis identified 7950 proteins, including 1192 core proteins shared by all groups, primarily associated with maintaining physical barrier integrity, responses to oxidative stress, and pathogen recognition. We identified 1328 juvenile-specific proteins enriched in the organophosphate metabolic process, cytoskeletal protein binding and hydrolase activity, complement and coagulation cascades, and phagosome pathways, whereas 833 adult-specific proteins were associated with macromolecule catabolic process, NOD-like (Nucleotide-binding Oligomerization Domain-like) receptor signaling, chemokine signaling, leukocyte transendothelial migration and Toll-like receptor signaling. This may indicate that juvenile tilapia skin mucus is characterized by high metabolic activity and innate immune effectors for broad-spectrum defense against environmental microbes, while adults employ integrated innate-adaptive mechanisms for targeted pathogen clearance. Following *C. cichlidarum* infection, the markedly increased abundance of 1343 proteins in tilapia skin mucus delineates a highly coordinated defense network. Functional enrichment analysis revealed that these proteins are primarily associated with catalytic activity and are significantly involved in key signaling pathways, including phagosome, C-type lectin receptor signaling, PI3K-Akt signaling, chemokine signaling, and the complement and coagulation cascades. Moreover, 64 *C. cichlidarum*-derived proteins were identified in the mucus of infected tilapias, including HSC (heat shock cognate protein) 70, serine/threonine protein phosphatase, calmodulin, and thioredoxin peroxidase. These proteins are potentially involved in the host invasion or immune evasion of *C. cichlidarum*. These results provide insights into the adaptability of fish mucosal immunity and suggest targets for future functional characterization.

## Simple Summary

Parasitic infections pose a significant economic challenge to tilapia aquaculture. The mucosal layer covering the fish’s epithelium acts as the first line of defense against pathogens such as parasites. This study examined changes in the protein profile of the tilapia mucosal layer during ontogenetic development and following parasitic infection. The aim was to determine how defensive adaptations vary across life stages. The analyses indicated that the mucosal immunity of juveniles emphasizes rapid, generalized barrier functions, whereas adults deploy a more specialized and targeted defense system. During parasite infection, surface mucus initiates a robust counter-response while concurrently activating regulatory pathways to mitigate excessive inflammation and self-damage. Furthermore, parasite-derived proteins were found in the mucus of infected tilapia, which may facilitate parasite colonization and suppress host fish immune recognition. This study reveals, for the first time, the protein composition of skin mucus from tilapia under different physiological states, providing fundamental data for studies on parasite–host immune interactions and offering new insights into the prevention and control of fish parasitic diseases.

## 1. Introduction

The Nile tilapia (*Oreochromis niloticus*) is a globally significant aquaculture species, listed by the Food and Agriculture Organization (FAO) as being important for freshwater production. As the world’s third most farmed fish, with an annual yield exceeding 4.5 million tons, it generates considerable economic value worldwide [1,2]. However, parasitic diseases pose a significant threat to the health and sustainability of the industry. These infections can cause mass fry mortality, stunted growth and direct physical damage, thereby reducing market quality and causing substantial economic losses for producers [3].

Monogeneans are small, hermaphroditic platyhelminths that exhibit strong host specificity and primarily parasitize the gills, skin and fins of teleost fish [4,5,6]. They usually attach via chitinous anchors, causing mechanical damage that leads to bleeding, ulceration and tissue necrosis; severe infections can be fatal [7,8]. These parasites feed on epithelial cells, mucus and blood from the host. In tilapia, *Cichlidarus cichlidarum* (synonym: *Gyrodactylus cichlidarum*) and *Cichlidogyrus* spp. (cichlid gill flukes) are particularly prevalent and harmful [9,10]. Studies confirm that the anchoring and migration of *C. cichlidarum* significantly damage the skin and gill tissues of hosts, inducing local inflammation. Infected fish exhibit behavioral signs of stress, alongside a significant upregulation of immune-related genes aimed at clearing the parasite [10].

The skin mucus of fish constitutes the primary interface with the aquatic environment, providing a multifaceted defense through physical, biochemical, and physiological mechanisms [11]. Fish cutaneous mucus is secreted by mucous cells extensively distributed in the epidermal tissue and on the gill surface. Its primary components are mucins and fibrous materials. Upon release from mucous cells, these secretions hydrate and expand upon contact with water, forming a gelatinous matrix that coats the free surface of the epithelium and constitutes a natural physical and immunological barrier. This gelatinous matrix, composed of water and mucins, forms a physical barrier that hinders pathogen adhesion, while its continuous secretion and shedding help to clear microbes from the epithelial surface [11]. Fish epidermal mucus contains numerous innate immune components, such as glycoproteins, lysozyme, complement proteins, lectins, ApeC-containing proteins, C-reactive proteins, flavoenzymes, proteolytic enzymes, and antimicrobial peptides, as well as immunoglobulins [12,13]. Studies have demonstrated that the innate immune factors in fish skin mucus can effectively kill parasites. For instance, the skin mucus of *Siganus oramin* has been shown to possess significant lethal effects against *Cryptocaryon irritans* and *Ichthyophthirius multifiliis*, with treated parasites exhibiting ciliate shedding, swelling, rupture of the outer membrane, and leakage of internal contents [14]. Similarly, the skin mucus of *Amphiprion clarkii* demonstrated strong antiparasitic activity against *C. irritans* theronts in a dose- and time-dependent manner. Microscopic observation revealed that treated theronts rapidly ceased swimming, followed by cilia shedding, cell rounding, membrane rupture, and content leakage [15]. Patel reported that the epidermal mucus of *Clarias batrachus* exhibits antipathogenic activity, with its mucus extract demonstrating significant inhibitory effects against *Escherichia coli* and *Staphylococcus aureus* [16].

To counteract host surface defense mechanisms, parasites employ specific molecules within their excretory/secretory proteins (ESPs) to achieve bidirectional regulation of host physiological and immune responses, thereby enhancing their own survival [17]. For example, certain parasite-derived adhesion proteins bind specifically to host epithelial receptors, facilitating attachment [18], whereas other secreted factors degrade defensive barriers in the host to facilitate tissue invasion and nutrient acquisition. Secretory proteins from the labial glands of salmon lice (*Lepeophtheirus salmonis*) interfere with the host’s inflammatory responses and inhibit coagulation, thereby promoting the establishment of the parasite [19]. The surface mucus of fish contains various humoral immune components, including complement, lysozyme, and antibodies, which can directly attack the parasite’s tegument [20]. In response to this immune pressure, monogeneans utilize their ESPs, including stefins [21], serpins [22], and kunitz domain proteins [23] to counteract host defenses. These inhibitors are considered key tools employed by parasites to evade host immunity by modulating host immune signaling pathways, thereby facilitating survival at the infection site. In vitro studies have demonstrated that a recombinant kunitz-type protein from *Eudiplozoon nipponicum* (rEnKT1) compromises both hemostasis and complement activation through the inhibition of Factor Xa, plasmin, and plasma kallikrein, consequently promoting the survival of *E. nipponicum* [17,23].

We hypothesize that the protein composition of fish skin mucus varies across developmental stages and in response to parasitic challenges, reflecting distinct modes of mucosal immune responses and, to some extent, elucidating the mechanisms underlying parasite–host mucosal interactions. In this study, we employed parasitic challenge experiments coupled with liquid chromatography–mass spectrometry (LC-MS) to characterize the proteomic profiles of skin mucus in juvenile and adult *O. niloticus*, as well as in *O. niloticus* following *C. cichlidarum* infection. Our objective is to provide foundational data elucidating the developmental characteristics of fish mucosal immunity and the mechanisms underlying parasite–host mucosal interactions, thereby identifying novel targets for the development of biomarkers and vaccines against fish parasitic diseases.

## 2. Materials and Methods

### 2.1. Fish and Parasites

All the experimental tilapias were purchased from Tilapia Breeding Center of Guangdong province (Guangzhou, China). The fish were acclimated in a laboratory recirculating aquaculture system at a temperature of 25 °C. During the acclimation period, they were examined to ensure they were free of parasite infection. Ultraviolet (UV) light was used to sterilize the circulating water system to avoid bacterial infection, and the filter material was cleaned periodically.

*Cichlidarus cichlidarum* for infection experiments was taken from a laboratory-maintained population, which had previously been established on the host *O. niloticus* [10,23]. The monogenean parasite *C. cichlidarum* was propagated on tilapia (body length: 3–4 cm) and subsequently collected for experimental infection. Tilapias (body weight: 1.8 ± 0.6 g; body length: 3.7 ± 0.5 cm, values are shown as means ± standard deviation) for parasite maintenance were cultured in the indoor experimental fish tank with recirculating freshwater at 25 °C without feeding. Subsequently, they were injected with 50–100 µL Dexamethasone Sodium Phosphate Injection-BL-DM11 (BOLAI YAOYE, Jiujiang, China) to allow a higher intensity of *C. cichlidarum* when they were infected with 5–10 individuals of *C. cichlidarum*. The parasite burden of fish was individually examined under a stereomicroscope (SZX16, Olympus, Tokyo, Japan)after 10 s anesthesia in solution of 500 mg/l ethyl 3-aminobenzoate methanesulfonate salt (A5040, Sigma-Aldrich, St. Louis, MO, USA). Subsequently, fins infested with *C. cichlidarum* were excised to harvest *C. cichlidarum* for the infection experiment [24].

Prior to the infection experiment, the healthy tilapias were examined under a stereomicroscope to confirm the absence of parasites and physical abnormalities. Five selected fish (body weight: 1.0 ± 0.6 g; body length: 3.7 ± 0.6 cm) were individually housed in 350 mL plastic containers for a 72 h acclimatization period, during which the water was changed daily. For infection, each fish was exposed to fin fragments carrying *C. cichlidarum* at a dose of approximately 100 parasites per fish. After 24 h post-infection, the fish were anesthetized with ethyl 3-aminobenzoate methanesulfonate and examined under a stereomicroscope to determine the parasite load. The intensity of parasites per fish were 52, 58, 52, 40, and 75, respectively. Subsequently, skin mucus was collected from these infected fish to serve as samples for the infected group.

### 2.2. Mucus Collection

Mucus samples were collected from five healthy juvenile tilapias (body weight: 1.0 ± 0.6 g; body length: 3.7 ± 0.6 cm), five infected tilapias (body weight: 1.0 ± 0.6 g; body length: 3.7 ± 0.6 cm), and three adult tilapias (body weight: 365.6 ± 62.6 g; body length: 27.0 ± 1.4 cm). The body surface of each fish was wiped with sterile absorbent cotton (HQH776, LEAGUE, Jiaozuo, China), which was then immersed in a 1.5 mL centrifuge tube containing Phosphate-Buffered Saline (PBS; pH 7.2–7.4, 8 mM Na_2_HPO_4_, 2 mM NaH_2_PO_4_, and 137 mM NaCl). Mucus samples from each group were pooled into one tube per group. After brief vortexing, the cotton was compacted using forceps, and the tubes were centrifuged at 8000× *g* for 5 min at 4 °C. The supernatant was then transferred to a new tube. Total protein concentration was determined using the Bicinchoninic Acid Assay (BCA), strictly following the manufacturer’s instructions for the BCA Protein Assay Kit (T9300A, Takara, Dalian, China). Subsequently, proteins were separated by sodium dodecyl sulfate–polyacrylamide gel electrophoresis (SDS-PAGE).

Due to the limited volume of skin mucus secreted by individual juvenile and infected tilapia, which yielded insufficient protein for individual LC-MS/MS analysis, samples from individuals within each experimental group were pooled. Prior to pooling, individual samples were assessed via SDS-PAGE to ensure biological consistency and similar protein profiles across individuals within the same group. Each pooled sample contained approximately 100 μg of total protein.

### 2.3. Mass Spectrometry

The gel fragments containing target proteins (approximately 1 mm^3^) were destained until colorless, washed with ultrapure water, dehydrated with acetonitrile until they turned white, and air-dried. The reduction process entailed the addition of 10 mM dithiothreitol (DTT) (incubated at 56 °C for 1 h), followed by alkylation with 55 mM IAM in the dark for 45 min. Following a series of washes and dehydrations, the gel pieces were then rehydrated in a trypsin solution (0.01 μg/μL in 25 mM ammonium bicarbonate (NH_4_HCO_3_)) on ice for a period of 30 min. This was followed by the addition of buffer in order to cover the pieces, and the incubation of the samples was carried out overnight at 37 °C. Peptides were extracted with acetonitrile, and the resulting upper layer was collected by means of centrifugation and lyophilization.

The lyophilized peptide samples were then reconstituted in mobile phase A (2% acetonitrile (ACN), 0.1% formic acid (FA)), clarified by centrifugation, and separated using an UltiMate 3000 ultra-high-performance liquid chromatography (UHPLC) system (Thermo Fisher Scientific, San Jose, CA, USA). The samples were initially loaded onto a trap column for the purpose of enrichment and desalting. Thereafter, separation occurred on a self-packed C18 column (75 μm inner diameter, 3 μm particle size, 25 cm length) at a flow rate of 300 nL/min with an effective gradient: 0–5 min, 5% B; 5–45 min, 5–25% B; 45–50 min, 25–35% B; 50–52 min, 35–80% B; 52–54 min, 80% B; 54–60 min, 5% B (B: 98% ACN, 0.1% FA). The LC system was connected to a Q-Exactive HF X (Thermo Fisher Scientific, San Jose, CA, USA) mass spectrometer via a nano electrospray ionization (ESI) source (voltage: 1.9 kV). The data were acquired in data-dependent acquisition (DDA) mode. Full-scan MS spectra (350–1500 m/z) were acquired at a resolution of 60,000, followed by higher-energy collisional dissociation (HCD) fragmentation of the top 30 precursor ions (charge 2+–6+, intensity threshold > 10,000) with MS/MS resolution of 15,000. The dynamic exclusion parameter was set to 30 s, and the automatic gain control (AGC) targets were set to 3E6 for MS and 1E5 for tandem mass spectrometry (MS/MS).

### 2.4. Protein Identification

The protein identification workflow began with the analysis of tandem mass spectrometry data using Mascot v2.3.02. The raw data were converted into peak lists and then searched against the protein database restricted to *Oreochromis niloticus* (GCA_001858045.3, https://asia.ensembl.org/Oreochromis_niloticus/Info/Index; accessed on 1 July 2026), and the data from infected tilapia group was also searched in the *C. cichlidarum* database (National Center for Biotechnology Information (NCBI) BioProject: PRJNA1229812). The search results were then filtered and subjected to quality control based on several established metrics (Table 1). All results were processed using Percolator [25] and filtered at a 1% spectrum-level false discovery rate (FDR; PSM-level FDR ≤ 0.01). The unique spectrum distribution, representing the non-redundant spectra from the identified peptides, was then assessed in order to evaluate the depth of the data acquisition. Identification confidence was gauged by the number of unique peptides—sequences that map uniquely to a single protein—with multiple unique peptides per protein increasing reliability. Protein sequence coverage, indicating the percentage of the protein sequence covered by identified peptides, reflected the comprehensiveness of the identification process. For quantitative analysis, peptide extracted ion chromatograms (XICs) were generated with BGI’s in-house software, and peptide peak areas were calculated. Protein abundances were then estimated using the intensity-based absolute quantification (iBAQ) algorithm, which sums the peptide peak areas for each protein and divides them by the number of theoretically observable peptides for that protein, yielding iBAQ values [26]. Finally, the filtered protein list was functionally annotated using gene ontology (GO) and Kyoto Encyclopedia of Genes and Genomes (KEGG) pathway analyses.

## 3. Results

### 3.1. The Proteins in Tilapia Mucus Identified by MS

A total of 2771, 2276, and 2858 proteins were identified in the juvenile, adult, and infected groups, corresponding to 16,279 (out of 65,490 total spectra), 11,158 (out of 65,715 total spectra), and 15,411 (out of 66,280 total spectra) matched spectra, respectively (Table 2).

The quality metrics demonstrated consistency across the groups. The distribution of unique spectra was comparable among groups, with a median of 2 and a mode of 1. The average protein sequence coverage was 5% for all groups. The ratios of multi-peptide to single-peptide identifications were also comparable, with values of 1.56 in juveniles (1688 out of 2771 proteins), 1.34 in adults (1305 out of 2276 proteins), and 1.47 in infected fish (1699 out of 2858 proteins).

### 3.2. Proteomic Profiling of Skin Mucus in Tilapia

**Figure 1:**
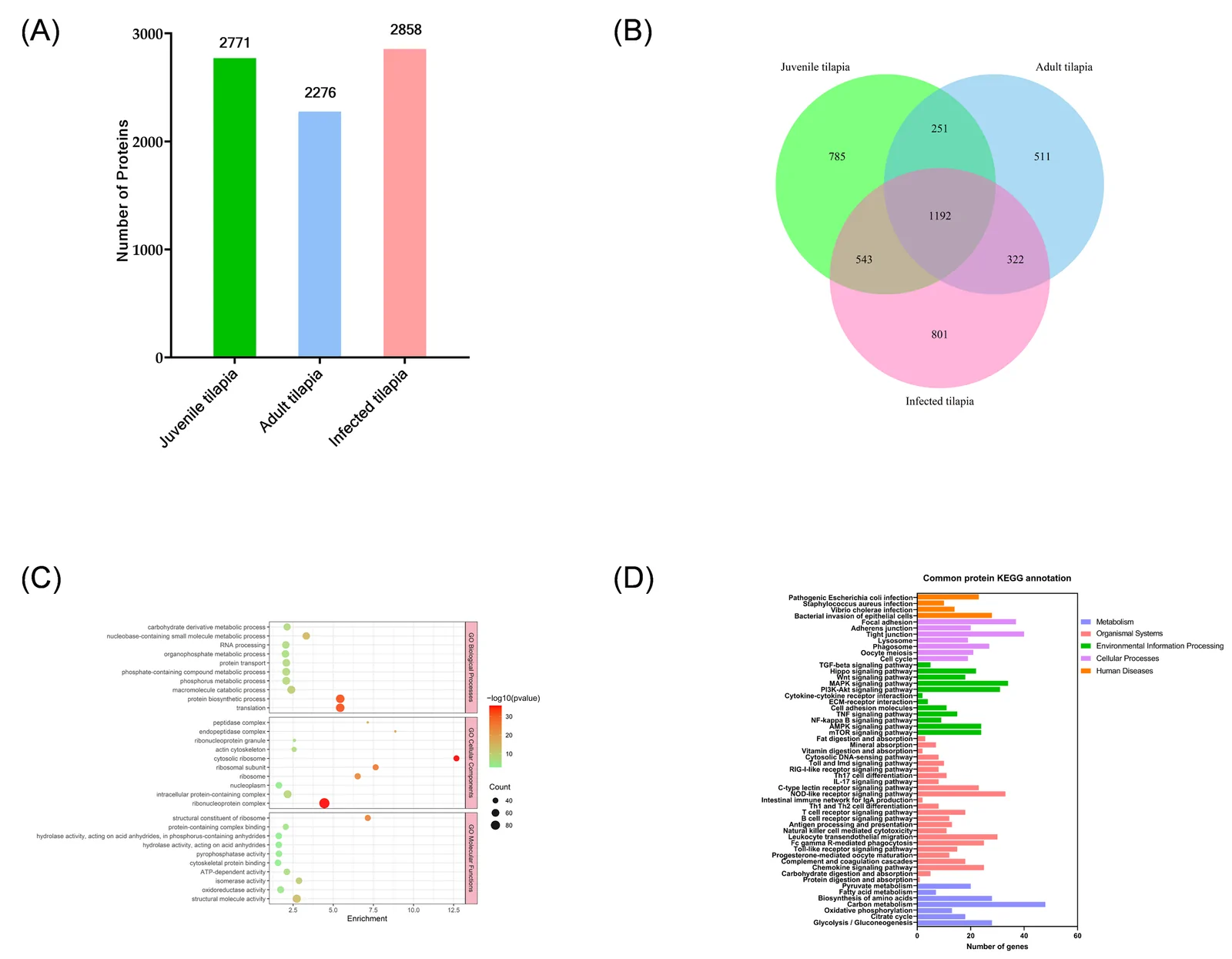
Identification and functional enrichment analysis of the skin mucus proteome in tilapia. (**A**) Number of identified proteins in the skin mucus of tilapia across the three groups. (**B**) Venn diagram depicting the number of unique and shared proteins identified in the skin mucus of juvenile, adult, and infected tilapia. (**C**) Gene ontology (GO) functional enrichment analysis of the common proteins identified in all three groups, displaying enriched terms within the categories of biological process (BP), cellular component (CC), and molecular function (MF). (**D**) Kyoto Encyclopedia of Genes and Genomes (KEGG) pathway enrichment analysis of the common proteins, illustrating the enriched biological pathways.

A total of 7905 proteins were identified in the skin mucus of tilapia, comprising 2771 in juveniles, 2276 in adults, and 2858 in infected tilapia (Figure 1A, Table S1). Among all the identified proteins, 785 were specifically detected in juvenile tilapia, 511 in adult tilapia, and 801 in infected tilapia (Figure 1B, Table S2). Furthermore, 1192 proteins (Table S2) were found to be common across all three groups (Figure 1B), and these proteins were enriched in functional categories including protein synthesis, primary energy metabolism, and antioxidant defense, as revealed by functional enrichment analysis. Analysis by gene ontology (GO) of the 1192 common proteins revealed that abundant proteins are associated with GO terms related to protein biosynthetic process, translation, ribonucleoprotein complex, intracellular protein-containing complex, structural molecule activity and oxidoreductase activity (Figure 1C). KEGG pathway analysis demonstrated that the common proteins were enriched in pathways such as carbon metabolism, Tight junction, Focal adhesion, MAPK signaling, NOD-like receptor signaling and C-type lectin receptor signaling pathways (Figure 1D). Among the common proteins, those with higher abundance include MHC class II antigen alpha, cystatin, serpin, transmembrane p24 trafficking protein, galectin, peroxiredoxin, C-type lectin, thioredoxin, complement C3, fibrinogen C-terminal domain-containing protein, endoplasmic reticulum protein 44 and prolyl endopeptidase, which contribute to promoting inflammation, counteracting oxidative stress, and safe-guarding immune cells from oxidative damage (Table 3).

### 3.3. Compositional Differences of the Skin Mucus Proteome in Tilapia Across Developmental Stages

**Figure 2:**
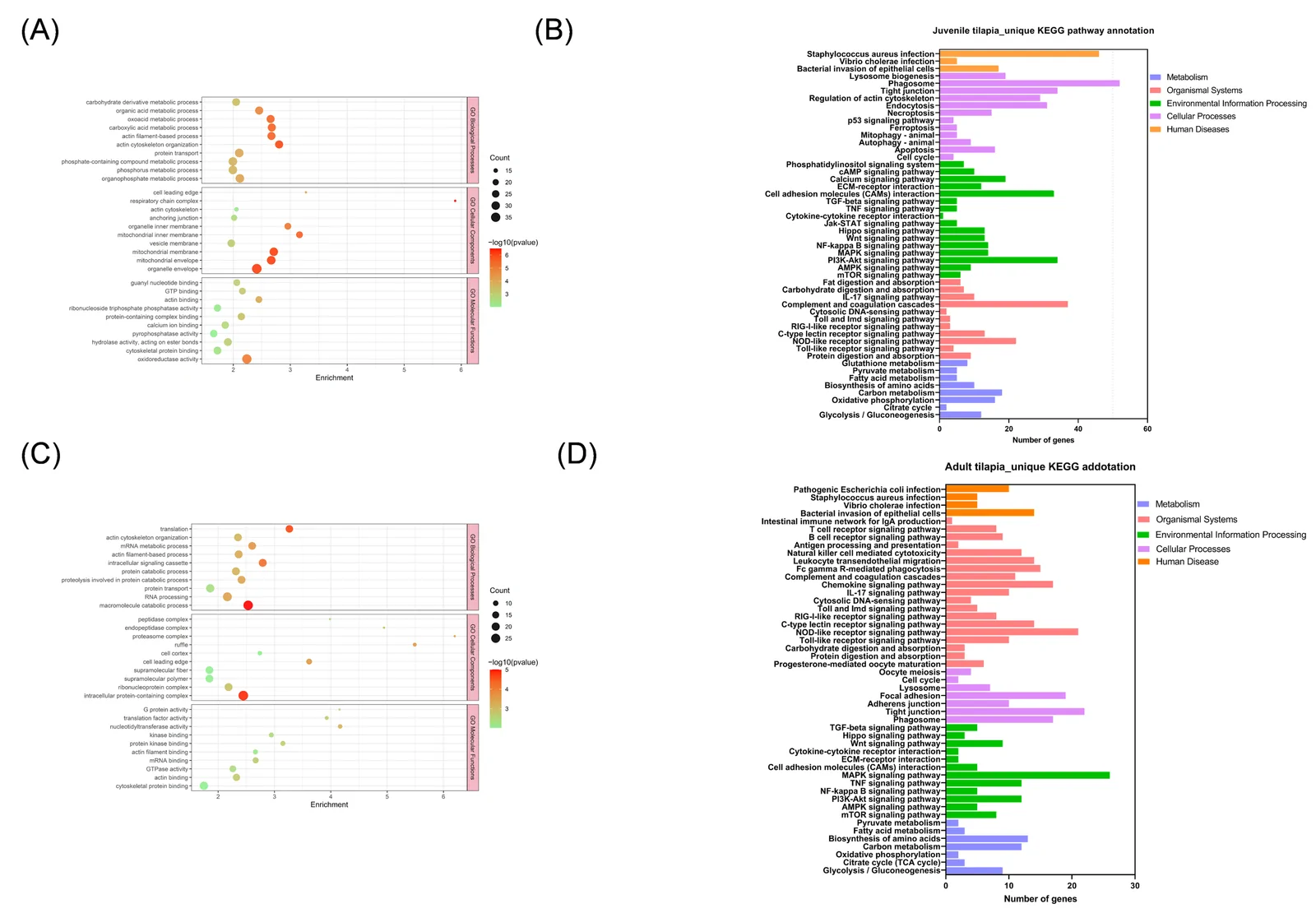
GO functional and KEGG pathway enrichment analyses of unique skin mucus proteins in juvenile and adult tilapia. (**A**) GO enrichment analysis of immune-related and growth and development proteins unique to juvenile tilapia; (**B**) KEGG pathway enrichment analysis of unique proteins in juvenile tilapia; (**C**) GO enrichment analysis of immune-related and growth and development proteins unique to adult tilapia; (**D**) KEGG pathway enrichment analysis of unique proteins in adult tilapia.

A comparative analysis was performed on the proteins identified from juvenile and adult tilapia groups: 1328 proteins were found to be unique to the juvenile group, whereas 833 proteins were uniquely detected in the adult group (Table S3). GO enrichment analysis demonstrated that the 1328 proteins specific to juvenile tilapia were primarily involved in the organophosphate metabolic process, actin cytoskeleton organization, organelle envelope, mitochondrial envelope, oxidoreductase activity, cytoskeletal protein binding and hydrolase activity (Figure 2A); and KEGG pathway enrichment analysis revealed these proteins were overrepresented in *Staphylococcus aureus* infection, Phagosome, cell adhesion molecule (CAM) interaction, complement and coagulation cascades, and PI3K-Akt signaling pathways (Figure 2B).

In comparison, the 833 proteins specific to adult tilapia were predominantly enriched in macromolecule catabolic process, RNA processing, intracellular protein-containing complex, ribonucleoprotein complex, cytoskeletal protein binding and actin binding (Figure 2C); and KEGG pathway enrichment analysis revealed that enrichment of these proteins in MAPK signaling, Tight junction, Focal adhesion, NOD-like receptor signaling, Chemokine signaling, Leukocyte transendothelial migration and Toll-like receptor signaling pathways (Figure 2D).

### 3.4. C. cichlidarum Induced Reprogramming of the Skin Mucus Proteome in Juvenile Tilapia

**Figure 3:**
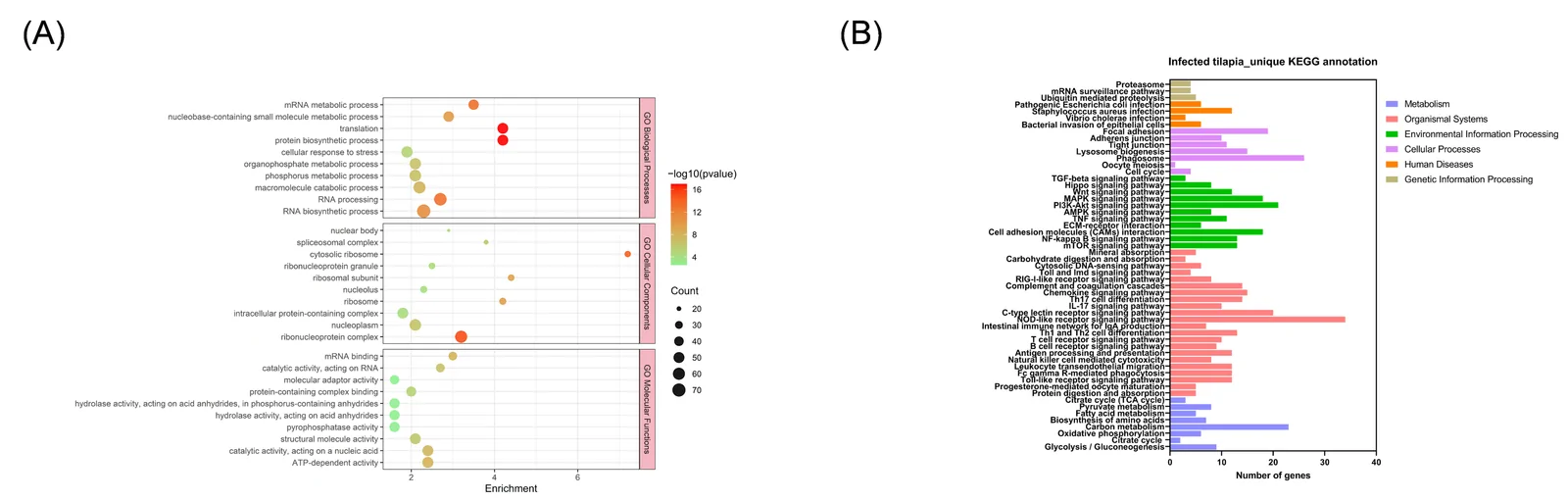
GO functional and KEGG pathway enrichment analyses of skin mucus proteins in infected tilapia. (**A**) GO functional classification of unique and upregulated proteins in infected tilapia; (**B**) KEGG pathway enrichment analysis of unique and upregulated proteins in infected tilapia.

A comparative analysis was performed on the proteins identified from juvenile and infected tilapia groups. 1123 proteins (Table S4) were uniquely detected in the infected group, and 220 proteins (Table S5) exhibited markedly increased abundance in the infected group compared with the juvenile group, as determined by iBAQ-based quantification (log_2_ fold change > 1). The unique and upregulated proteins mainly include the immunoglobulin M (IgM) variable region of the heavy-chain (VH) region, complement C3a, complement C9, ubiquitin-conjugating enzyme, integrin α2, interferon-induced protein 35, calreticulin, apoptotic protease-activating factor 1, proteasome subunit β, vacuolar Protein Sorting 35 (VPS35) endosomal protein-sorting factor, paxillin, fibronectin type-III, MHC II β, granzyme K, interstitial collagenase, selectin E, metalloendopeptidase, lectin mannose-binding 2, sestrin 2, C-type lectin, cathepsin B, gelsolin, keratin and serpin (Table 4). These proteins are mainly involved in RNA biosynthetic process, protein biosynthetic process, ribonucleoprotein complex, adenosine triphosphate (ATP)-dependent activity, catalytic activity and structural molecule GO terms (Figure 3A); and in phagosome, NOD-like receptor signaling, C-type lectin receptor signaling, Focal adhesion, PI3K-Akt signaling, carbon metabolism, MAPK signaling, chemokine signaling, and complement and coagulation cascades pathways (Figure 3B).

### 3.5. C. cichlidarum-Derived Proteins in the Skin Mucus of Infected Tilapia

**Figure 4:**
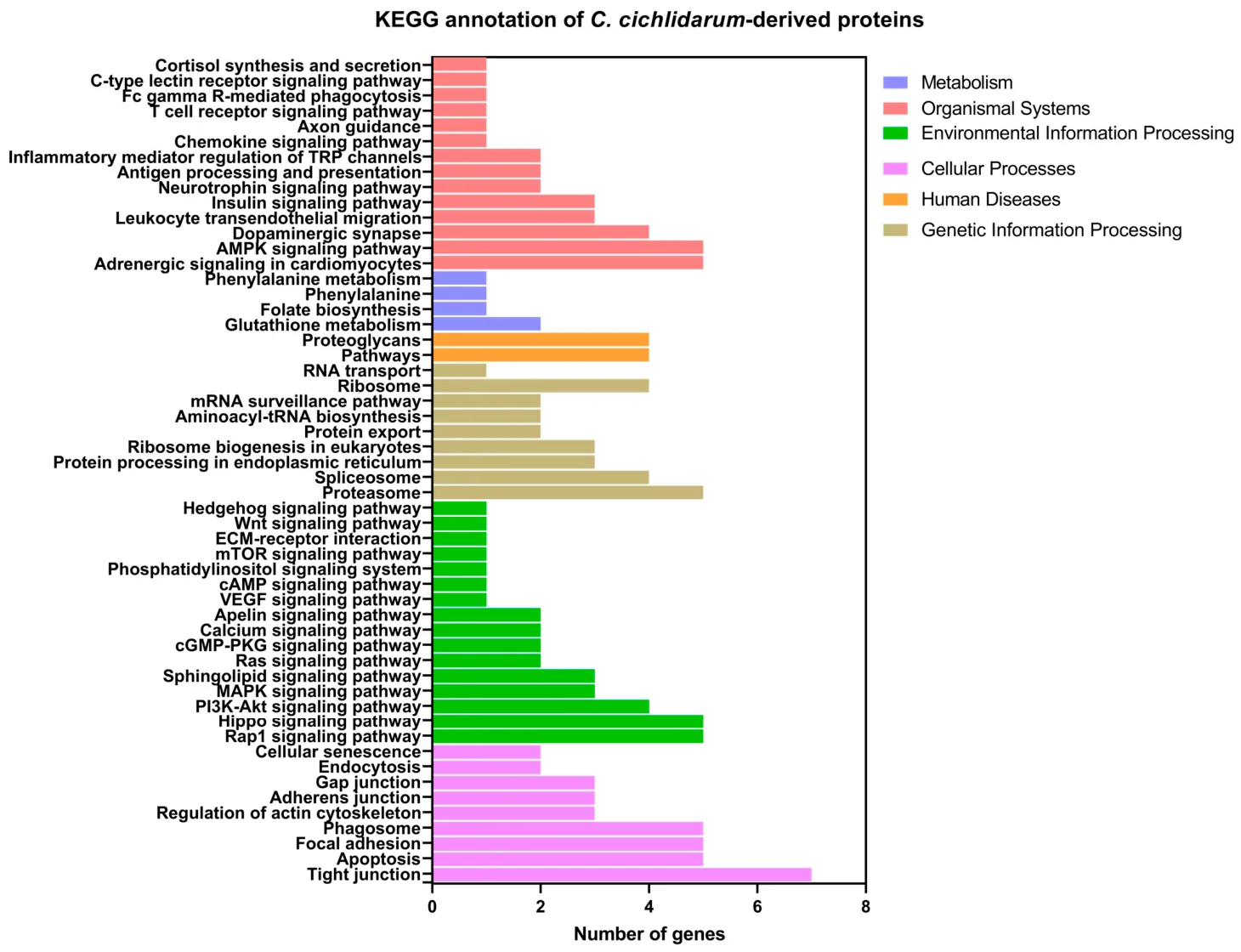
GO functional enrichment analyses of *C. cichlidarum*-derived proteins.

Database-specific protein identification revealed that 64 proteins from the skin mucus of infected tilapia were uniquely assigned to the *C. cichlidarum* database (Table S6). KEGG pathway enrichment analysis revealed these proteins were overrepresented in Tight junction, Rap1 signaling, Focal adhesion, proteasome, Adrenergic signaling in cardiomyocytes, Hippo signaling and PI3K-Akt signaling pathways (Figure 4). The enrichment of these pathways reflects the strategies employed by *C. cichlidarum* to adapt to the host environment and sustain its survival and proliferation.

From the 64 *C. cichlidarum*-derived proteins, 17 proteins are potentially involved in immune evasion or host–parasite interactions, mainly including myosin, actin (cytoplasmic), calmodulin, heat shock cognate protein (HSC70), tubulin, thioredoxin peroxidase, and serine/threonine-protein phosphatase (Table 5).

## 4. Discussion

The skin mucus of teleost fish acts as a primary physicochemical barrier against pathogens and contains various immune components, including immunoglobulins and enzymes. This study utilized comparative proteomics to analyze skin mucus from juvenile, adult and infected tilapias, thereby elucidating the dynamic regulation of this mucosal barrier during host ontogeny and parasitic challenge.

The 1192 proteins common to all three experimental groups constitute the core mucosal proteome, underpinning essential physiological functions required for developmental stability and environmental adaptation. Tilapia mucus functions as both a physical barrier and a dynamic biochemical interface, integrating redox homeostasis, innate immune recognition, and antigen presentation, thereby counteracting environmental stressors and pathogen invasion. Enrichment of proteins associated with protein biosynthetic processes, translation, and ribonucleoprotein complexes was observed. This profile suggests a high potential for protein synthesis, which may support the continuous renewal of the mucus layer through the secretion of nascent mucins and antimicrobial peptides [90]. Additionally, the presence of proteins involved in Tight junction and Focal adhesion pathways suggests the presence of structural components necessary for epithelial barrier integrity. These components are known to maintain tissue mechanical stability and restrict paracellular pathogen penetration in teleosts, potentially preventing secondary bacterial infections during parasitic infestation [91]. Maintaining the integrity of these junctional structures is particularly critical during parasitic infection, as barrier disruption can facilitate the penetration of secondary pathogens into deeper tissues [92]. The enrichment of NOD-like receptor and C-type lectin receptor (CLR) signaling pathways, along with the high abundance of C-type lectins, suggests that tilapia mucus possesses the molecular machinery for pathogen recognition. C-type lectins are known to recognize surface carbohydrates and can initiate downstream signaling, contributing to antibacterial defense [45,93]. Similarly, the high abundance of Complement C3 implies the capacity for complement-mediated immune responses, such as opsonization and pathogen lysis, although functional activation status requires further biochemical verification [34]. Moreover, the detection of MHC class II antigen alpha in skin mucus is not exclusive to tilapia; this molecule was also identified in the skin mucus proteome of *Anguilla anguilla* infected with Anguillid herpesvirus 1 [94]. This cross-species presence suggests that fish skin mucus universally possesses the molecular basis for local antigen presentation. While MHC class II is typically expressed by professional antigen-presenting cells, its presence in the mucus may derive from infiltrating immune cells or, potentially, from epithelial cells under specific conditions, thus facilitating an interface with adaptive immunity [35]. The high abundance of peroxiredoxin and thioredoxin, alongside the enrichment of GO term oxidoreductase activity, suggests the existence of a robust antioxidant defense network within the mucus. This observation is consistent with findings from proteomic and peptidomic studies of epidermal mucus in two Chilean aquaculture species, *Seriola lalandi* and *Seriolella violacea*, in which abundant antioxidant enzymes mitigate oxidative stress induced by elevated seawater oxygen tension and pathogen-triggered reactive oxygen species (ROS) bursts [95,96]. By scavenging ROS, these enzymes protect immune cells from oxidative injury and sustain their functional competence [44,46]. This antioxidant mechanism is particularly critical during parasitic infection, as host–parasite interactions often trigger intense inflammatory responses and ROS production; excessive oxidative stress can subsequently cause tissue damage and immunosuppression [97]. Therefore, the elevated abundance of these proteins may represent an adaptive response to mitigate oxidative injury in tilapia mucus, potentially helping to maintain immune effector functions within a regulated range. Additionally, the high abundance of cystatin and serpin suggests the tight regulation of protease activity by the mucosa. During infection, parasites such as *Ichthyophthirius multifiliis* secrete proteases that degrade the mucosal barrier, thereby facilitating their invasion into host tissues [98]. The presence of cystatin and serpin in the mucus suggests a host strategy to inhibit these exogenous proteases and modulate endogenous proteases, thereby potentially preventing tissue damage caused by excessive inflammation. This balance is critical for limiting parasite invasion and mitigating pathological damage. Additionally, the presence of galectin and fibrinogen C-terminal domain-containing proteins (FReDs) contributes to the immune effector repertoire of mucus. Galectin participates in cell adhesion and immune regulation, whereas FReDs are implicated in wound healing and inflammatory responses [29,42].

Proteins showing compositional differences in the skin mucus between juvenile and adult tilapia suggest an ontogenetic shift in the mucosal immune system, moving from metabolically driven nonspecific innate defenses toward receptor-mediated specific immunity, while maintaining barrier integrity. This finding not only elucidates the dynamic remodeling of teleost skin as a primary immune barrier but also offers evidence for the evolutionary conservation of the mucosal immune system across vertebrates [99]. In juvenile fish, skin mucus proteins were enriched in GO terms associated with organophosphate metabolic processes, mitochondrial envelope components, oxidoreductase activity, and hydrolase activity, as well as in KEGG pathways associated with *Staphylococcus aureus* infection, Phagosome, complement and coagulation cascades, and the PI3K-Akt signaling pathway. This enrichment pattern implies a dual reliance on high metabolic energy and basal innate immunity to sustain rapid somatic development. The elevated abundance of proteins associated with the mitochondrial envelope and organophosphate metabolism suggests that mucosal epithelial cells in juvenile fish are highly metabolically active, which may support the energy demands for mucus secretion, rapid epithelial turnover, and antimicrobial factor synthesis [45]. The abundance of oxidoreductases and hydrolases may contribute to a constitutive chemical barrier, potentially providing broad-spectrum and immediate antimicrobial protection by generating ROS or directly degrading pathogen cell walls [96]. This pathogen recognition-independent defense mechanism may be particularly important for juvenile fish, given their immature immune systems and limited adaptive immune memory. Furthermore, the pronounced enrichment of complement and coagulation cascades highlights the potential importance of humoral immunity in juvenile fish. As a key effector mechanism of innate immunity, the complement system facilitates phagocytic clearance of pathogens via opsonization [34]. The enrichment of the phagosome pathway further suggests a reliance of juvenile fish on phagocyte-mediated, nonspecific clearance mechanisms against environmental microbial challenges [100]. Moreover, the enrichment of the PI3K-Akt pathway, beyond its canonical roles in cell survival and proliferation, may be involved in maintaining epithelial integrity and promoting wound healing, which could be advantageous for juvenile fish vulnerable to physical injury [45]. Additionally, cell adhesion molecule (CAM) interactions may facilitate rapid epithelial cell migration and tissue repair [96]. In contrast, the skin mucus proteins of adult fish were enriched in GO terms related to macromolecule catabolic, RNA processing, and ribonucleoprotein complexes, as well as in KEGG pathways associated with MAPK signaling, Tight junction, Focal adhesion, NOD-like receptor signaling, chemokine signaling, leukocyte transendothelial migration, and Toll-like receptor signaling. This enrichment pattern suggests a transition toward a more complex and regulated immune strategy. The enrichment of TLR and NOD-like receptor signaling pathways indicates the presence of a mature pattern recognition receptor (PRR) network in the adult skin mucosa, facilitating the recognition of pathogen-associated molecular patterns (PAMPs) and the initiation of inducible immune responses [101,102]. This receptor-based defense mechanism may allow adult fish to mount more targeted immune responses, potentially limiting tissue damage caused by excessive inflammation [103]. As a key downstream signaling hub, the MAPK pathway transduces PRR signals to regulate gene expression, thereby coordinating the release of inflammatory mediators [104]. Furthermore, enrichment of the Tight junction and Focal adhesion pathways suggests that adult fish possess molecular adaptations for maintaining physical barrier integrity. With increasing body size and prolonged environmental exposure, preserving epithelial Tight junction is critical for resisting pathogen invasion and sustaining homeostasis [105]. The enrichment of chemokine signaling and leukocyte transendothelial migration pathways suggests a robust immune cell recruitment mechanism in the adult skin mucosa, potentially facilitating the mobilization of circulating immune cells, such as lymphocytes and macrophages, to infection sites [106]. Together with humoral immunity, this cell-mediated immune response may contribute to a more comprehensive defense system in adult fish. Notably, the presence of proteins linked to TCR and BCR signaling pathways in adults suggests a greater involvement of adaptive immune mechanisms at the mucosal surface compared to juveniles. In teleosts, immunoglobulin T (IgT, also known as immunoglobulin Z (IgZ) in some species) serves as the antibody isotype specifically mediating mucosal immunity. Through convergent evolution, IgT has acquired functional properties analogous to those of mammalian immunoglobulin A (IgA), including the regulation of mucosal microbiota homeostasis and defense against pathogen invasion [107]. IgT is predominantly secreted by mucosal-associated B cells. Enrichment of BCR signaling components suggests that the adult skin mucosa harbors a functional adaptive immune compartment capable of antigen recognition, although further functional assays are needed to confirm antigen-specific activation [108]. In contrast, the lower enrichment of this pathway in juvenile fish suggests that the immune system may rely primarily on nonspecific innate defenses, including the complement system and phagocytosis, rather than established adaptive memory [45]. This developmental disparity aligns with the broader paradigm of vertebrate immune ontogeny, wherein innate immunity confers immediate post-hatching protection, whereas adaptive immunity necessitates gene rearrangement, clonal selection, and functional maturation.

Proteomic analysis of skin mucus from tilapia following *C. cichlidarum* infection revealed that 1343 proteins exhibited markedly increased abundance, with enriched pathways involved in inflammatory response, cytoskeletal reorganization, complement activation, and antigen presentation. These changes may collectively contribute to a multi-layered defense network against ectoparasite infestation [45]. The concurrent upregulation of CLR signaling components and mannose-binding lectin 2 (MBL2) is consistent with the recognition of parasite surface glycans [43,44]. As pattern recognition receptors, C-type lectins specifically recognize and bind carbohydrate structures on pathogen surfaces [45], which may initiate downstream immune signaling and activate complement and coagulation cascades, as evidenced by the markedly increased abundance of complement components such as C3a and C9 [75,109]. C3a, a potent anaphylatoxin, recruits immune cells to sites of infection, whereas C9, a core component of the membrane attack complex (MAC), has the potential to mediate pathogen lysis [78]. Furthermore, enrichment of the NOD-like receptor signaling pathway suggests the potential detection of parasite-associated molecular patterns by intracellular sensors, which may lead to inflammasome assembly and pro-inflammatory cytokine release [110]. Although the skin mucosa is generally considered a primary effector site of innate immunity, the present study detected upregulation of the IgM heavy-chain VH region and MHC class II β-chain in the skin mucus proteome of infected tilapia, indicating active adaptive immune participation at the mucosal surface [111]. IgM, the predominant immunoglobulin in teleost serum and mucosa, showed upregulation in its VH region, suggestive of a specific antibody response to parasite antigens [73]. The upregulation of MHC class II molecules and IgM heavy-chain VH regions is associated with potential enhancements in antigen presentation and mucosal antibody responses. These findings imply the involvement of local antigen-presenting cells in processing parasite antigens, a process that potentially contributes to the initiation of adaptive immune memory [36]. This interplay between innate and adaptive immune components may contribute to host defense against parasitic infections. The increased abundance of proteins associated with PI3K-Akt and MAPK signaling pathways suggests their potential involvement in supporting immune cell functions and metabolic demands during infection [112,113]. Concurrently, elevated expression of the Focal adhesion pathway and its associated proteins—including integrin α2, paxillin, and fibronectin type III—implicates enhanced epidermal cell–extracellular matrix interactions [56,58,63]. Such cytoskeletal modifications may contribute to the maintenance of epithelial barrier integrity, wound healing, and immune cell migration [114]. Given that parasite attachment can damage the epithelium, integrin α2-associated signaling may participate in subsequent tissue repair processes, representing a potential host defense mechanism [63]. The enrichment of the phagosome pathway, along with the elevated abundance of ubiquitin-conjugating enzymes, proteasome subunit β, and the endosomal sorting factor VPS35, suggests that the host may enhance intracellular degradation mechanisms to clear phagocytosed parasite debris and damaged cellular components [60]. The ubiquitin–proteasome system tags and degrades abnormal or foreign proteins, thereby generating peptide fragments for antigen presentation [62]. VPS35, a core component of the retromer complex, mediates endosomal cargo sorting and recycling, ensuring proper localization of immune receptors and signaling molecules [55]. In addition, upregulation of cathepsin B and granzyme K suggests the potential activation of lysosome-mediated killing and cytotoxic responses, which may contribute to the degradation of parasite structural proteins or the induction of apoptosis in infected cells [50,70]. Detection of interferon-induced protein 35 and Sestrin 2 indicates host responsiveness to oxidative stress and inflammation induced by parasitic infection [48,79]. Sestrin 2, a stress-responsive protein, regulates AMP-activated protein kinase (AMPK) and mammalian target of rapamycin (mTOR) signaling to balance energy metabolism and autophagy [79]. The enrichment of the carbon metabolism pathway suggests that metabolic reprogramming may occur to meet the elevated energy demands of immune cells during infection [115]. Increased abundance of keratin and serpin suggests a potential strengthening of the epithelial barrier, which may help limit further pathogen invasion and mitigate tissue damage caused by excessive protease activity [41,67]. Thus, skin mucus proteins with markedly increased abundance following infection may serve as candidate biomarkers for the non-invasive diagnosis of gyrodactyliasis.

Notably, several *C. cichlidarum*-derived proteins were identified in the skin mucus of infected tilapia. Based on previous studies in other parasites, these proteins are inferred to be involved in host invasion or immune evasion [81,116]. For example, as a member of the HSP (Heat Shock Protein) 70 family, *Schistosoma mansoni* HSC (heat shock cognate protein) 70 has been shown in recent studies to interact with immune receptors of the snail host *Biomphalaria glabrata*, potentially suppressing stress signaling and facilitating immune evasion [82,83,117]. This interaction can suppress host stress signaling and immune defenses or dampen pro-inflammatory immune responses, thereby facilitating immune evasion [118]. Serine/threonine kinases and phosphatases jointly regulate critical cellular processes, including cell-cycle progression, transcription, metabolism, cytoskeletal dynamics, motility, apoptosis, and differentiation [85,86], and they have been demonstrated to be key regulators of parasite growth, development, reproduction, and immune modulation. In *Schistosoma japonicum*, the catalytic subunit of serine/threonine protein phosphatase 1 (PP1) is essential for worm growth, pairing stability, and reproductive development [85]. Similarly, serine/threonine protein phosphatase of *Eimeria tenella* (EtSTP) shows high expression during developmental stages and directly regulates the invasion process [119]. In *Haemonchus contortus*, Ehsan et al. identified HcSTP-1 as a key effector molecule mediating host immunosuppression by directly binding to host immune cells, thereby inhibiting their proliferation and antigen presentation [120]. Calmodulin, a ubiquitous and highly conserved eukaryotic Ca^2+^ sensor, orchestrates cell-cycle progression, signal transduction, and motility by triggering the activation of downstream kinases and phosphatases following calcium binding [80,121]. Calcium signaling is a key driver of parasite movement and host cell invasion. For example, the calcium-binding protein TgpCaBP in *Toxoplasma gondii* is essential for its motility, invasion, and secretion [80]. In *Schistosoma mansoni*, the calcium-binding protein SmE16 is highly expressed at the host–parasite interface and responds to calcium signals [84]. Thioredoxin peroxidase (Prx/TPx) serves as a core antioxidant enzyme that not only scavenges H_2_O_2_ through a thioredoxin (Trx)-dependent catalytic cycle but also participates in the regulation of intracellular redox signaling [122]. At the host–parasite interface, thioredoxin peroxidase functions as a key immune evasion strategy; for instance, *Schistosoma japonicum* Prx-1 effectively clears ROS generated by both parasite metabolism and host immune cells, thereby providing direct protection against oxidative damage [123]. Furthermore, these proteins function as dual-purpose immunogens that modulate host immune responses, *Toxoplasma gondii* peroxiredoxin 3 (TgPrx3) is recognized by the host immune system and stimulates the production of the pro-inflammatory cytokine IL-12, thereby exhibiting both antigenic and immunomodulatory properties [124]. In summary, these proteins play a crucial role in the host invasion and survival of parasites, and they may serve as promising targets for drug and vaccine development.

Constrained by multiple factors, sufficient mucus protein samples were not available to establish biological replicates at the outset of this study. Consequently, only a single pooled sample per treatment group was subjected to LC–MS analysis, representing a major limitation of this study. In the absence of biological replication, we could not determine whether the observed differences in protein abundances across treatment groups were statistically significant. Thus, we restricted our analysis to simple comparisons of total protein abundances among groups. Therefore, our primary focus was on comparing protein composition across treatment groups under consistent analytical sensitivity—specifically, identifying proteins detected universally across all groups versus those detected exclusively in specific groups. It is important to note that these results are influenced by inter-individual variability and entail a non-negligible false-positive rate. Accordingly, the findings should be regarded as preliminary and hypothesis-generating. Guided by these observations, we will undertake rigorous quantitative and functional studies on selected proteins derived from both the host fish skin mucus and the parasite.

## 5. Conclusions

The interaction between parasitic invasion and host immune defense represents a dynamic interplay, with skin mucus serving as the primary interface where this molecular crosstalk initiates. This study reveals that the core proteome of *Oreochromis niloticus* skin mucus is primarily associated with maintaining physical barrier integrity, responding to oxidative stress, and facilitating pathogen recognition. Compared to juveniles, adult tilapias exhibit a more diverse repertoire of pathogen recognition receptors (PRRs) and proteins linked to adaptive immunity in their skin mucus. Following *Cichlidarus cichlidarum* infection, there was a marked increase in the abundance of proteins involved in catalytic activity, phagosome formation, C-type lectin receptor signaling, PI3K-Akt signaling, chemokine signaling, and the complement and coagulation cascades. Furthermore, we identified several parasite-derived proteins in the mucus of infected fish, including HSC70, serine/threonine protein phosphatase, modulation, and thioredoxin peroxidase, which may play critical roles in parasite invasion and immune evasion. The identification of these proteins further opens new possibilities for targeted interventions against monogenean parasites, which remain a significant threat to global tilapia production.

## Figures and Tables

**Table 1:** Mascot search parameters.

Item	Value
Search Engine	Mascot v2.3
Enzyme	Trypsin
Peptide Mass Tolerance	20 ppm
Fragment Mass Tolerance	0.05 Da
Fixed modifications	Carbamidomethyl (C)
Variable modifications	Oxidation (M); Gln->pyro-Glu (N-term Q); Deamidated (NQ)
Max Missed Cleavages	1
Instrument type	ESI-FTICR
Database	*Oreochromis_*nr.fasta (Supplementary File S1, 127,678 sequences) *C. cichlidarum*_nr.fasta (Supplementary File S2, 21,331 sequences)

**Table 2:** Protein identification results.

Sample	Total Spectra	Identified Spectra	Identified Peptides	Identified Proteins
Juvenile tilapia	65,490	16,279	12,801	2771
Adult tilapia	65,715	11,158	9124	2276
Infected tilapia	66,280	15,411	12,382	2858

**Table 3:** Consensus highly abundant proteins identified in skin mucus of tilapia across three groups.

Protein ID	Protein	Q-Score	Function	iBAQ	Reference
A0A668SX24_OREAU	Endoplasmic reticulum protein 44	35.134785	ER resident folding assistant	2321785	[27]
A0A668VBK2_OREAU	Prolyl endopeptidase	12.346393	Specific hydrolysis of proline-containing substrates.	401751	[28]
I3KSA2_ORENI	Fibrinogen C-terminal domain-containing protein	15.857026	Participates in the coagulation cascade and exerts hemostatic effects by forming a fibrin clot.	4853431	[29]
A0A669F087_ORENI	Calpain catalytic domain-containing protein	67.965228	Calcium-dependent cysteine protease superfamily, participated in the regulation of key physiological processes, such as gene expression, cytoskeleton remodeling, and apoptosis.	2944150	[30]
A0A668TU60_OREAU	Prenylcysteine oxidase 1	8.8260929	Participates in the degradation of isoprenylated proteins, catalyzes the cleavage of thioether bonds, releasing hydrogen peroxide, cysteine, and isopentenal, thereby regulating oxidative stress and protein homeostasis.	216777.9	[31]
A8WE65_ORENI	Glutathione S-transferase	28.945713	Acts as a multifunctional phase II detoxification enzyme, catalyzing the conjugation of glutathione with endogenous and exogenous compounds, and participating in steroid hormone metabolism and xenobiotic detoxification.	13682315	[32]
A0A668TNP6_OREAU	Superoxide dismutase [Cu-Zn]	21.142701	Destroys radicals that are normally produced within the cells and are toxic to biological systems.	2572170	[33]
A0A669CZZ2_ORENI	Complement C3	48.919128	Key components of the lectin pathway of complement activation.	954759.7	[34]
A0A3S5GZG0_ORENI	MHC class II antigen alpha	5.2856753	Adaptive immune antigen presentation.	24281947	[35]
A0A668VQ59_OREAU	14-3-3 protein	49.243473	Maintains mucosal barrier integrity and coordinates local immune responses.	15039232	[36]
I3K0F6_ORENI	Coatomer subunit gamma	79.30494	Participation in vesicular trafficking and protein retrieval, and maintenance of ER-Golgi homeostasis.	14722351	[37]
A0A668RD68_OREAU	Cystatin	29.509696	Responds to bacterial infection, participates in host defense, suppresses pathogenic bacteria, and modulates inflammatory responses.	12183077	[38]
A0A668RLL4_OREAU	Cytokine receptor-like factor 3	31.855954	Negatively regulates antiviral immunity, promotes viral replication and immune evasion.	9872161	[39]
A0A668S9I0_OREAU	Serpin	48.113301	Participates in host anti-pathogen immune responses, suppresses inflammatory responses, regulates the complement system, and inhibits serine protease activity.	9759930	[40]
A0A668S833_OREAU	Transmembrane p24 trafficking protein	7.3963523	Mediates vesicular transport and acts as a cargo receptor for specific protein transport.	9555165	[41]
A0A668T8H9_OREAU	Galectin	28.327944	Agglutinates and directly kills pathogens, participates in pro-inflammatory or anti-inflammatory responses, promotes cell migration, and mediates intercellular recognition and immune signaling initiation.	8098435	[42]
A0A669E432_ORENI	Macrophage-capping protein	66.113242	Regulates actin filament polymerization, organization, and localization, and modulates cell motility and morphological changes.	8070420	[43]
A0A668UBL5_OREAU	Peroxiredoxin	44.976262	Participates in protection against oxidative stress and regulation of H_2_O_2_ signaling, and possesses multiple functions including chaperone and phospholipase activities.	8018155	[44]
A0A669AVK5_ORENI	C-type lectin	12.732848	Recognizes and binds pathogen glycans in a calcium-dependent manner, and participates in mucosal defense and complement activation.	5747206	[45]
A0A668U664_OREAU	Thioredoxin	7.999464	Participates in anti-oxidative stress and regulates the transcription of inflammatory factors and immune-related genes.	4764323	[46]

**Table 4:** The unique and upregulated proteins in the skin mucus of infected tilapia.

Protein ID	Protein	Q-Score	Function	iBAQ	Reference
A0A669EIY0_ORENI	NLR family pyrin domain-containing 12	4.225972347	A NOD-like receptor (NLR) family member that inhibits NF-κB activation, activates the inflammasome, and facilitates immune cell migration.	62711.5991	[47]
A0A669DZF8_ORENI	Interferon-induced 56 kDa protein	4.003868502	Capable of mediating protein–protein interactions and participating in host immune defense as well as exerting antiviral effects	4438956.28	[48]
A0A669DJ15_ORENI	Fish-egg lectin	2.197220598	A fish-specific pattern recognition receptor (PRR) and core innate immune recognition factor with bacterial binding and agglutination activities as well as NF-κB inhibitory function.	113898.286	[49]
I3K9V2_ORENI	Granzyme K	2.009332085	Promotes inflammatory responses; regulates apoptosis and pyroptosis via activation of JAK-STAT, MAPK, and NF-κB signaling pathways.	296454.624	[50]
A0A668UFE7_OREAU	Calreticulin	27.68259317	It exhibits both bacterial binding and agglutination activities, thereby contributing to antimicrobial immunity.	642638.361	[51]
A0A669D2M2_ORENI	Cathepsin L1	3.023802303	Participates in the innate immune response to bacterial infection.	343195.733	[52]
H9B8H8_ORENI	MHC class II beta	2.514967036	Adaptive immune antigen presentation	322268.103	[53]
A0A669B9J3_ORENI	Selectin E	2.109726398	Participates in inflammatory responses.	32777.26	[36]
I3KFV5_ORENI	Interstitial collagenase	5.267365032	Participates in tissue repair by mediating the degradation and remodeling of extracellular matrix collagen.	285202.955	[54]
A0A669FCS9_ORENI	VPS35 endosomal protein-sorting factor	15.30143978	Acts as a core component of the retromer complex and mediates the transport of specific proteins.	1506278.17	[55]
A0A668U5P8_OREAU	Paxillin	13.77920355	Acts as a key adaptor protein in focal adhesions and regulates cytoskeletal dynamics as well as the dynamic assembly and disassembly of focal adhesions.	1048792.42	[56]
A0A668U7T6_OREAU	P32	15.8020951	Participates in immune defense against bacterial infection, binds C1q, and modulates immune responses.	767544	[57]
I3IWM8_ORENI	Fibronectin type III	19.87760436	Acts as a structural spacer or as a platform for mediating protein–protein interactions.	312781.1	[58]
A0A668TIQ1_OREAU	Mitogen-activated protein kinase kinase kinase 15	28.73711221	Participates in osmotic stress responses, regulates apoptosis, and maintains tissue homeostasis.	89541.051	[59]
A0A668TTG1_OREAU	Proteasome subunit β	17.82903549	Acts as the core catalytic component of the 20S proteasome, and the conserved threonine residue within its catalytic domain is essential for proteolysis.	13099782.4	[60]
A0A669E9Z9_ORENI	GLI pathogenesis-related 2	28.24753758	Negatively regulates autophagy.	9081116.18	[61]
A0A669DX69_ORENI	Ubiquitin-conjugating enzyme	12.83958034	Transfers activated ubiquitin molecules from the ubiquitin-activating enzyme to the ubiquitin ligase, thereby mediating the ubiquitination of target proteins.	4724409.21	[62]
A0A669D6P3_ORENI	Integrin α2	49.68509483	Mediates cell adhesion and migration, and participates in the regulation of innate immunity and autoimmunity.	975291.441	[63]
I3JTN9_ORENI	Interferon-induced protein 35	13.07825826	Participates in regulating the production of pro-inflammatory cytokines and IFN-proteasome subunit-β, enhances interferon and inflammatory responses, and positively regulates host innate immune responses to suppress viral replication.	622811.342	[64]
I3JZT5_ORENI	Apoptotic protease-activating factor 1	5.267365032	Determines, through differential ligand binding, whether cells undergo intrinsic stimuli-activated apoptosis or NF-κB-driven inflammation.	21757.4483	[65]
A0A669BZ75_ORENI	Gelsolin	117.5126831	Acts as a highly conserved actin-binding protein, with core functions of binding, severing, and capping actin filaments.	21377371.7	[66]
A0A668T0J2_OREAU	Keratin	83.11788013	Provides mechanical strength, participates in building physical barriers, and promotes cell adhesion and proliferation.	12805117.4	[67]
A0A668SC02_OREAU	Heat shock protein	2.146289441	Acts as a highly conserved molecular chaperone that binds and assists in the intracellular folding, assembly, transport, or degradation of peptides.	11695688.3	[68]
I3IY14_ORENI	Serpin	51.61511878	Participates in host anti-pathogen immune responses, suppresses inflammatory responses, regulates the complement system, and inhibits serine protease activity.	10959787.7	[69]
A0A669CN52_ORENI	alpha-1,2-Mannosidase	2.480409162	Acts as a key enzyme in glycoprotein processing and maturation by catalyzing the trimming of N-linked glycans on glycoproteins.	6020522.84	[41]
I3KKC0_ORENI	Cathepsin B	13.26880361	Exhibits both exonuclease and endonuclease activities and plays important roles in physiological processes including antigen presentation, degradation, apoptosis, inflammatory responses, and various diseases.	4232655.43	[70]
A0A669DHH8_ORENI	Calpain catalytic domain-containing protein	25.77834515	Calcium-dependent cysteine protease superfamily, participated in the regulation of key physiological processes, such as gene expression, cytoskeleton remodeling, and apoptosis.	3201898.12	[71]
A0A669BH40_ORENI	Thioredoxin	13.57160646	Participates in anti-oxidative stress and regulates the transcription of inflammatory factors and immune-related genes.	2544170.54	[46]
A0A668ST15_OREAU	Mannose-6-phosphate isomerase	7.527932729	Catalyzes the reversible isomerization between mannose-6-phosphate and fructose-6-phosphate, and serves as a pivotal enzyme in carbohydrate metabolic pathways.	2224437.82	[47]
A0A669F3Z6_ORENI	Serine protease	2.559958327	Acts as an endopeptidase that specifically cleaves internal peptide bonds, plays important roles in the complement system, and participates in inflammatory responses.	852107.159	[72]
A0A126CP29_ORENI	IgM heavy-chain VH region	4.003868502	Possesses highly specific antigen-binding capability.	682716.078	[73]
I3J411_ORENI	TNF receptor-associated factor	3.329143038	Acts as a key adaptor protein in innate immune signaling pathways and belongs to the E3 ubiquitin ligase family.	591894.489	[74]
I3JYR1_ORENI	Complement component C9	17.38851081	Participates in the formation of the membrane attack complex on the surface of target cells and plays an important role in defense against invading pathogens.	294816.274	[75]
A0A669CQC8_ORENI	C-type lectin	2.514967036	Recognizes and binds pathogen glycans in a calcium-dependent manner, and participates in mucosal defense and complement activation.	140309.91	[76]
I3KHP2_ORENI	Metalloendopeptidase	2.081472031	Belongs to the metalloendopeptidase family, possesses proteolytic activity, and participates in apoptosis, angiogenesis, tissue repair, and immune responses.	129374.469	[45]
A0A669B2J4_ORENI	Complement subcomponent C1r	7.06388878	Serves as a recognition subunit of the complement system, binds to C1r and C1s, and plays a key role in pathogen clearance through the classical complement pathway.	128256.808	[77]
A0A669DJ38_ORENI	Lectin, mannose-binding 2	3.734745742	Acts as a key protein involved in innate immune responses, recognizes specific molecular patterns, and activates the lectin pathway of the complement system.	127412.029	[78]
A0A669EU04_ORENI	Complement C3a	16.6376047	Acts as a key inflammatory mediator and chemokine, mediates the recruitment of immune cells to the site of infection, enhances inflammatory responses, and promotes cytokine secretion.	91145.601	[45]
A0A668RS24_OREAU	Sestrin 2	5.267365032	Acts as a stress-inducible metabolic regulator and a conserved antioxidant protein.	1661.06806	[79]

**Table 5:** *C. cichlidarum* derived from proteins associated with immune evasion or host interaction.

Protein ID	Protein	Q-score	Function	iBAQ	Reference
Gene.36647::Unigene1584_GC1::g.36647::m.36647	Myosin motor domain-containing protein	5.750077935;	A myosin family component that, together with actin, constitutes the coiled elastic muscle assembly responsible for parasite body extension, flexion, and elastic recoil.	100823.9705;	[65]
Gene.1487::CL124.Contig1_GC1::g.1487::m.1487	Myosin-3	3.64642996		18928.7977	
Gene.33215::CL4363.Contig2_GC1::g.33215::m.33215	Actin-related protein isoform 3	3.646429958;	Actin family proteins that assemble into actin bands and cooperate with myosin to form elastic muscle fibers, endowing the parasite body with contractile properties.	860289.9621;	[65]
Gene.19418::CL1793.Contig3_GC1::g.19418::m.19418	Actin;	10.93928988;		164423.9659;	
Gene.42190::Unigene11657_GC1::g.42190::m.42190	Actin, cytoplasmic	7.29285992		3028532.56	
Gene.33784::CL4573.Contig1_GC1::g.33784::m.33784	Calmodulin	9.50926788	As a core molecule that activates downstream kinases and phosphatases following Ca^2+^binding, it regulates cell-cycle dynamics, signal transduction pathways, and motility, ultimately serving as the key driving force behind parasite motility and host invasion.	1556171.7	[80]
Gene.36326::Unigene1083_GC1::g.36326::m.36326	Heat shock cognate protein	3.64642996	By facilitating polypeptide folding and repairing stress-damaged proteins, this protein serves as a core regulator of cellular proteostasis.	253599.731	[81,82,83]
Gene.42883::Unigene12909_GC1::g.42883::m.42883	Thioredoxin peroxidase	3.64642996	By utilizing a thioredoxin (Trx) system-dependent catalytic cycle, this protein eliminates H_2_O_2_ and contributes to the modulation of intracellular redox signaling.	2427422.66	[84]
Gene.34170::CL4716.Contig1_GC1::g.34170::m.34170	Serine/threonine-protein phosphatase delta	2.216407967;	Involvement in the regulation of cell-cycle progression, transcription, metabolism, cytoskeletal rearrangement, motility, apoptosis, and differentiation.	34527.23403;	[85]
Gene.38704::Unigene5559_GC1::g.38704::m.38704	Serine/threonine-protein phosphatase alpha	7.29285992		174497.909	
Gene.34268::CL4756.Contig1_GC1::g.34268::m.34268	26S proteasome regulatory subunit6A,6B,7,8	7.292859917;	Component of the 26S proteasome; enables ATP binding, ATP hydrolysis, and proteasome-activating activities	192205.619;	[86]
Gene.41327::Unigene10023_GC1::g.41327::m.41327		5.862837926;		119026.937;	
Gene.40545::Unigene8686_GC1::g.40545::m.40545		3.646429958;		106813.1851;	
Gene.21505::CL2030.Contig2_GC1::g.21505::m.21505		18.2321498		715513.001	
Gene.8216::CL684.Contig4_GC1::g.8216::m.8216	Tubulin	3.64642996	Is a key component of the cytoskeleton, plays a critical role in almost all cellular and developmental processes, participates in membrane maturation, transport machinery, and secretory activities that support parasite survival both in vitro and in vivo, and is considered a potential drug target for treating parasitic infections.	1817806.82	[87]
Gene.40864::Unigene9092_GC1::g.40864::m.40864	Putative aminopeptidase	3.64642996	Participates in fundamental cellular and developmental processes, including the processing of nascent proteins, activation of zymogens and prohormones, and maintenance of metabolic homeostasis and clearance of cellular metabolites.	205758.862	[88]
Gene.27713::CL3012.Contig1_GC1::g.27713::m.27713	Casein kinase 1 alpha	3.64642996	Is a member of the casein kinase 1 family, participates in DNA processing and repair, cell division, and other cellular processes, is required for normal tissue regeneration and maintenance, and its knockdown results in reduced regeneration rate and morphological abnormalities in the head and tail.	332926.867	[89]

## Data Availability

The original mass spectrometry proteomics data have been deposited to the ProteomeXchange Consortium (https://proteomecentral.proteomexchange.org; accessed on 7 July 2026 ) via the iProX partner repository with the dataset identifier PXD080618.

## Supplementary Materials

The following supporting information can be downloaded at: https://www.mdpi.com/article/10.3390/biology15201864/s1, Supplementary File S1: *Oreochromis*_nr.fasta, 127,678 sequences; Supplementary File S2: *C. cichlidarum*_nr.fasta, 21,331 sequences; Table S1: Proteins derived from juvenile/adult/infected tilapia; Table S2: Group—specific and common proteins in juvenile, adult, and infected tilapia; Table S3: Stage—specific protein expression in juvenile and adult; Table S4: Unique proteins in infected tilapia; Table S5: Upregulated protein in infected tilapia; Table S6: *Cichlidarus cichlidarum*-derived proteins in infected tilapia.
